# Right-ventricular mechanics assessed by cardiovascular magnetic resonance feature tracking in children with hypertrophic cardiomyopathy

**DOI:** 10.1371/journal.pone.0248725

**Published:** 2021-03-18

**Authors:** Joanna Petryka-Mazurkiewicz, Lidia Ziolkowska, Łukasz Mazurkiewicz, Monika Kowalczyk-Domagała, Agnieszka Boruc, Mateusz Śpiewak, Magdalena Marczak, Grażyna Brzezinska-Rajszys

**Affiliations:** 1 Magnetic Resonance Unit, National Institute of Cardiology, Warsaw, Poland; 2 Department of Coronary and Structural Heart Disease, National Institute of Cardiology, Warsaw, Poland; 3 Department of Pediatric Cardiology, The Children’s Memorial Health Institute, Warsaw, Poland; 4 Department of Cardiomyopathy, National Institute of Cardiology, Warsaw, Poland; Faculty of Medical Science - State University of Campinas, BRAZIL

## Abstract

**Background:**

Although hypertrophic cardiomyopathy (HCM) is considered a disease of the left ventricle (LV), right ventricular (RV) abnormalities have also been reported on. Cardiovascular magnetic resonance feature tracking (CMR-FT) accurately and reproducibly quantifies RV myocardial deformation.

**Aim:**

To investigate RV deformation disorders in childhood HCM using CMR-FT.

**Material and methods:**

Consecutive subjects aged <18 years with echocardiographic evidence of HCM were enrolled. Cardiovascular magnetic resonance (CMR) was performed including RV volumetric and functional assessment, and late gadolinium enhancement (LGE) imaging.

**Results:**

We included 54 children (37 males, 68.5%) with HCM, of which 28 patients (51.8%; mean extent of 2.18 ± 2.34% of LV mass) had late gadolinium enhancement. LV outflow tract obstruction (LVOTO) was detected in 19 subjects (35.2%). In patients with LVOTO, RV global longitudinal strain (RVGLS) (-16.1±5.0 vs. -20.7±5.3, p<0.01), RVGLS rate (-1.05±0.30 vs. -1.26±0.40, p = 0.03), RV radial strain (RVR) (15.8±7.7 vs. 22.1±7.0, p<0.01) and RVR rate (0.95±0.35 vs. 1.6±0.44, p<0.01) were lower than in patients without LVOTO. The RVR rate (p<0.01) was lower in patients with LGE in comparison to patients without LGE.

**Conclusions:**

Children with HCM, especially with LVOTO, have significantly reduced indices of RV mechanics despite normal RV systolic function. It seems that the degree of LVOT obstruction is responsible for compromising the RV dynamics, rather than either mass or the amount of LV fibrosis.

## Background

Hypertrophic cardiomyopathy (HCM) is one of most common primary myocardial diseases in children, which can be characterized by massive myocardial proliferation, vast fibrosis and life-threatening prognosis [1, 2]. Although HCM is traditionally considered a disease of the left ventricle, right ventricular (RV) abnormalities have also been reported on [3–5], but not extensively characterized. It is believed that right ventricular involvement in LV pathologies is a consequence of a direct injury extension, afterload changes or ventricular interdependence, which is mainly due to the close anatomical association between the two ventricles. With myocardial deformation assessed by speckle-tracking echocardiography (STE) regional myocardial function can be quantified [6] and ventricular dysfunction may be detected in an earlier phase than with conventional echocardiography. The anatomical and functional RV assessment is deeply hampered in standard echocardiography mainly due to complex RV geometry, location and mechanics [7]. Previous STE-derived studies showed that right ventricular systolic function was impaired in HCM patients when compared with healthy subjects [8].

Cardiovascular magnetic resonance (CMR) offers better spatial resolution and visualisation of complex RV geometry than STE [9, 10]. Additionally, CMR may deliver a detailed assessment of the extent and distribution of both myocardial proliferation and fibrosis [11, 12]. Furthermore, feature tracking (FT) technique allows to quantify myocardial deformation from standard *cine* sequences without the need for additional tagged images [13, 14]. The link between decreased CMR-derived LV myocardial mechanics and the extent of hypertrophy and late gadolinium enhancement (LGE) has been reported on in childhood HCM [15]. Furthermore, the association of decreased LV myocardial mechanics by both two-dimensional echocardiography and CMR FT with an unfavorable prognosis has been found in adult HCM populations [16, 17]. However, very few research studies investigated how right ventricular (RV) myocardial mechanics are affected by cardiac hypertrophy in paediatric population. Thus, this study aims to apply feature tracking to investigate the alternations of right-ventricular deformation disorders, particularly with the relation to the extent of hypertrophy, degree of left ventricular outflow tract obstruction (LVOTO) and amount of LGE.

## Material and methods

### Ethics approval and consent to participate

The Institutional Ethics Committee of the Children’s Memorial Health Institute in Warsaw approved the study. A written informed consent to participate in the study was obtained from all patients and their parents.

We included consecutive subjects aged <18 years at the time of diagnosis with echocardiographic evidence of LV hypertrophy defined as diastolic LV wall thickness z-score ≥ 2 [determined as more than two standard deviations from the mean value for the population corrected for body surface area (BSA)] in the absence of hemodynamic conditions that could account for the observed hypertrophy [18]. The study also included patients with syndrome-associated HCM. The left ventricular outflow tract (LVOT) gradient of >30 mmHg was considered as significant. No child requiring sedation during CMR scan was enrolled in this study.

### CMR examination

CMR was performed on a 1.5-T scanner (Sonata and Avanto fit, Siemens, Germany). For volumetric and functional assessment electrocardiogram-gated, breath-hold, steady-state free precession cine images were acquired in standard orientations with a segmented k-space steady-state free-precession technique, using 25 phases per cardiac cycle. Late gadolinium enhancement (LGE) images were obtained in long-axis and short-axis imaging planes with a breath-hold segmented inversion recovery sequence performed 10–15 min after intravenous administration of 0.1 mmol/kg of gadobutrol (Gadovist, Bayer, Germany).

### Image analysis

Steady-state free precession (SSFP) images served for the calculation of ventricular volumes and ejection fraction with the use of dedicated software (MASS 6.2.1, Medis, Leiden, The Netherlands). Manual delineation of endocardial and epicardial contours was performed in end-diastolic and end-systolic phases. LV (LVEDV) and RV (RVEDV) end-diastolic volumes, LV (LVESV) and RV (RVESV) end-systolic volumes, LV (LVSV) and RV (RVSV) stroke volumes and LV (LVM) mass, as well as left (LVEF) and right (RVEF) ventricular ejection fractions were calculated. All volumetric parameters were indexed to the BSA. The papillary muscles were each time excluded from the LV mass calculation. The presence of left ventricular LGE was initially determined using visual assessment by two independent experienced observers and if positive, quantified. Quantification was performed with QMass v7.6 (Medis, Leiden, Netherlands) software as a signal intensity threshold of >6 SD above remote myocardium (>6 SD LGE). The extent of LGE was presented as the percentage of total LV mass.

### Feature tracking

**Fig 1:**
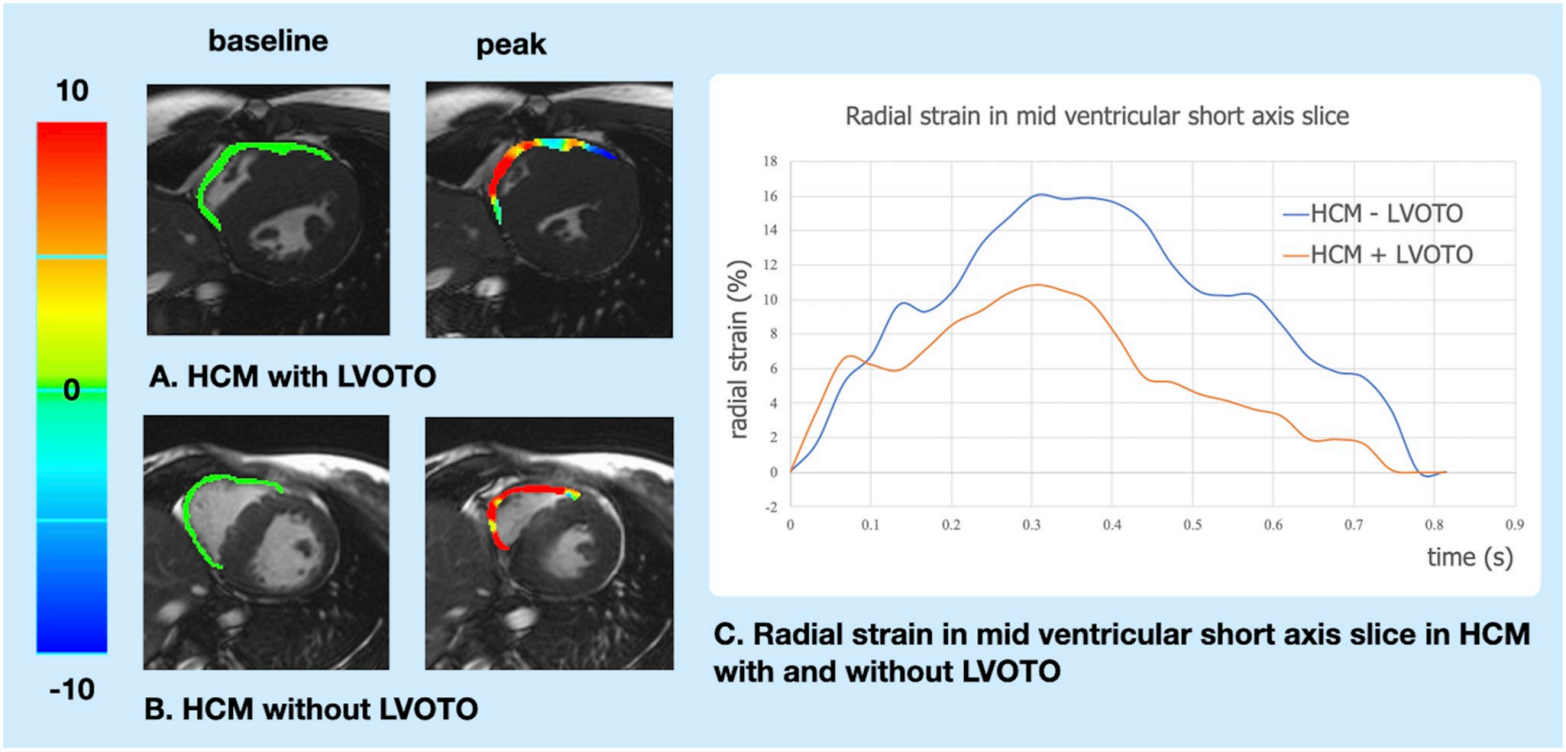
CMR feature tracking analysis of right ventricular radial strain in the mid short axis slice in patients with hypertrophic cardiomyopathy with and without left ventricular outflow tract obstruction. A. Color map of RV radial strain in a patient with LVOTO at baseline (left panel) and at peak (right panel). B. Color map of RV radial strain in a patient without LVOTO at baseline (left panel) and at peak (right panel). C. Curves of RV radial strain in the mid ventricular short axis slice in HCM with (red) and without (blue) LVOTO. CMR—cardiovascular magnetic resonance feature tracking, HCM—hypertrophic cardiomyopathy, LVOTO—left ventricular outflow tract obstruction, RV—right ventricle.

RV myocardial feature tracking analysis was performed based on the acquired b-SSFP cine images using a dedicated software (cvi42, Circle Cardiovascular Imaging Inc., Calgary, Alberta, Canada). The global right ventricular longitudinal peak strain (RVGLS) and global longitudinal peak strain rate (RVGLS rate) parameters were obtained from a 4-chamber view. Peak circumferential and radial strain and strain rates parameters (RVCS, RVCS rate, RVRS and RVRS rate, respectively) were determined in short axis view at the basal and mid-ventricular section of the ventricle. Because of the known tendency for artefacts and lower muscle thickness in the peri-apical slices, these slices were not included in the CMR-FT analysis. The basal slice in short axis view was defined as the first slice below the atrioventricular level showing a complete myocardial enclosing. The mid-ventricular slice was localized at the level of left ventricular papillary muscles. Endocardial and epicardial contours were drawn manually twice for each patient by two independent and skilled operators. Values of peak circumferential and radial strain and strain rates obtained in each slice of RV were averaged (Fig 1).

### Statistical analysis

All continuous variables were expressed as mean ± standard deviation (SD) or as median and interquartile range and were tested for normal distribution with the use of the Kolmogorov–Smirnov test. Comparisons between groups were performed using the Student’s t-test or Wilcoxon-Mann-Whitney U test for continuous variables, and chi-square or Fisher exact test for categorical variables as appropriate. Correlation analysis was performed using the Pearson’s method. Intra- and interobserver variability were evaluated using the Bland-Altman test and expressed as bias ± SD (95% confidence interval—CI) and intraclass correlation coefficients (ICCs), and the coefficient of variation (CoV). A two-sided *p*-value of less than 0.05 was considered to indicate statistical significance. Statistical analyses were performed with the use of MedCalc 12.1.4.0 software (MedCalc, Mariakerke, Belgium).

## Results

The study cohort comprised 54 children (37 males, 68.5%, mean age 12.03±4.71, mean BSA 1.3±0.46) with HCM, of which 30 (55.6%) children had family history of HCM. There was no hypertrophy of RV free wall found in the studied cohort. In the study group 19 subjects (35.2%) had LVOT obstruction (LVOTO). Expectedly, children with LVOTO were older than children without LVOTO (14.1±3.9 vs. 10.9±4.8, p = 0.01). There were no differences found in terms of right ventricular volumes and right ventricular ejection fraction (RVEF) between children with obstructive vs non-obstructive HCM (Table 1).

**Table 1:** CMR-derived right ventricular parameters in children with non-obstructive vs. obstructive hypertrophic cardiomyopathy.

	HCM without LVOTO, n = 35	HCM with LVOTO, n = 19	*p* value
Age (years)	10.9±4.8	14.1±3.9	0.01
Females (%)	13 (37%)	4 (21%)	ns
RVEDVI (mL/m^2^)	72.9±25.9	64.3±18.1	ns
RVESVI (mL/m^2^)	29.0±13.2	23.9±9.7	ns
RVSVI (mL/m^2^)	42.6±17.5	39.5±12.6	ns
RVEF (%)	60.8±8.5	62.2±6.7	ns
RVGLS (%)	-20.7±5.3	-16.1±5.0	<0.01
RVGLS rate (1/s)	-1.26±0.40	-1.05±0.30	0.03
RVCR (%)	-12.2±5.4	-13.2±3.8	ns
RVCR rate (1/s)	-0.95±0.43	-0.97±0.31	ns
RVR (%)	22.1±7.0	15.8±7.7	<0.01
RVR rate (1/s)	1.6±0.44	0.95±0.35	<0.01

CMR—cardiovascular magnetic resonance, HCM—hypertrophic cardiomyopathy, LVOTO—left ventricular outflow tract obstruction, RVEDVI—right ventricular end diastolic volume index, RVESVI—right ventricle end systolic volume index, RVSVI—right ventricular stroke volume index, RVEF—right ventricular ejection fraction, RVGLS—right ventricular global longitudinal strain, RVCR—right ventricular circumferential strain, RVR—right ventricular radial strain.

The RV longitudinal and radial strain and strain rate parameters varied depending on the presence of LVOT obstruction. In patients with hypertrophic obstructive cardiomyopathy (HOCM) the values of RVGLS (-16.1±5.0 vs. -20.7±5.3, p<0.01), RVGLS rate (-1.05±0.30 vs. -1.26±0.40, p = 0.03), RVR (15.8±7.7 vs. 22.1±7.0, p<0.01) and RVR rate (0.95±0.35 vs. 1.6±0.44, p<0.01) were significantly compromised when compared with subjects without LVOTO. The presence of LVOTO did not change the values of RV circumferential strain and strain rate (p = ns). The comparison of the indices of RV strains and strain rates in subjects with and without LVOTO is presented in Table 1.

As previously published [14], LGE was detected by quantitative analysis in 28 patients (51.8%). Among those, 12 children (43%) had fibrosis in RV/LV insertion points and in 16 children (57%) diffuse septal fibrosis was observed. The mean fibrosis extent in children with LGE was 2.18 ± 2.34% of LV mass. In patients with detected fibrosis on CMR, the RVR rate was significantly impaired compared to patients without LGE (1.14±0.40 vs. 1.61±0.5, p<0.01). Table 2 compares the indices of LV strains and strain rates in children with and without LGE.

**Table 2:** CMR-derived right ventricular parameters in children with vs. without late gadolinium enhancement.

	HCM with LGE, n = 28	HCM without LGE, n = 26	*p* value
Age (years)	12.9±4.7	11.0±4.6	ns
Females (%)	6 (21%)	11 (42%)	ns
RVEDVI (mL/m^2^)	65.4±21.2	74.6±25.6	ns
RVESVI (mL/m^2^)	25.0±11.8	29.7±12.6	ns
RVSVI (mL/m^2^)	39.8±13.3	43.3±18.3	ns
RVEF (%)	61.8±8.1	60.8±7.9	ns
RVGLS (%)	-17.7±6.1	-20.5±4.9	ns
RVGLS rate (1/s)	-1.16±0.38	-1.22±0.38	ns
RVCR (%)	-13.6±3.5	-11.4±5.9	ns
RVCR rate (1/s)	-0.92±0.26	-0.99±0.50	ns
RVR (%)	18.7±7.8	21.1±7.9	ns
RVR rate (1/s)	1.14±0.40	1.61±0.51	<0.01

CMR—cardiovascular magnetic resonance, HCM—hypertrophic cardiomyopathy, LGE—late gadolinium enhancement, RVEDVI—right ventricular end diastolic volume index, RVESVI—right ventricular end systolic volume index, RVSVI—right ventricular stroke volume index, RVEF—right ventricular ejection fraction, RVGLS—right ventricular global longitudinal strain, RVCR—right ventricular circumferential strain, RVR—right ventricular radial strain.

Furthermore, we found a negative correlation between RV radial strain and strain rate, and the degree of hypertrophy measured with LVMI (RVR p = 0.04 and RVR rate p < 0.01) (Table 3). Also, RVGLS rate correlated positively with LVMI (p < 0.01). While RVGLS (p = 0.02), RVR (p = 0.02) correlated positively with LVOT gradient, RVR rate correlated negatively with LVOT gradient (p < 0.01). None of the RV mechanical correlated with the extent of fibrosis.

**Table 3:** Pearson’s correlations between indices of right ventricular mechanics and left-ventricular outflow tract gradient, left-ventricular mass and amount of fibrosis.

Mechanical RV indices	LVOT gradient		LGE %		LVMI	
	Pearsons r	p	Pearsons r	p	Pearsons r	p
RVGLS (%)	0.31	0.02	0.16	ns	0.09	ns
RVGLS rate (1/s)	0.20	ns	-0.23	ns	0.44	<0.01
RVCR (%)	-0.13	ns	-0.25	ns	0.13	ns
RVCR rate (1/s)	-0.006	ns	-0.04	ns	0.13	ns
RVR (%)	0.31	0.02	-0.11	ns	-0.27	0.047
RVR rate (1/s)	-0.54	<0.01	-0.19	ns	-0.41	<0.01

LVOT—left ventricular outflow tract, LGE%–amount of late gadolinium enhancement as a percentage of left ventricular mass, LVMI—left ventricular myocardial mass index, RVGLS—right ventricle global longitudinal strain, RVCR—right ventricle circumferential strain, RVR—right ventricle radial strain.

The indices of reproducibility for right ventricular strain and strain rate parameters analysis were satisfactory—intraobserver and interobserver ICCs ranged between 0.83 and 0.99 for all RV components of myocardial performance (Table 4).

**Table 4:** Intra- and interobserver reproducibility of feature tracking.

	Intraobserver variability			Interobserver variability		
	Mean difference±SD	ICC (95%CI)	CoV (%)	Mean difference±SD	ICC (95%CI)	CoV (%)
RVGLS (%)	3.37±1.01	0.98 (0.96–0.99)	14.22	-4.75±1.58	0.96 (0.94–0.97)	17.8
RVGLS rate (1/s)	-0.23±0.09	0.96 (0.94–0.98)	16.57	-0.66±0.26	0.83 (0.73–0.90)	32.8
RVCR (%)	0.78±1.08	0.97 (0.88–0.99)	7.46	0.39±2.03	0.94 (0.89–0.96)	11.3
RVCR rate (1/s)	-0.07±0.18	0.87 (0.86–0.96)	13.59	0.16±0.17	0.93 (0.64–0.97)	18.77
RVR (%)	-2.14±0.93	0.99 (0.98–0.99)	7.96	-2.73±1.02	0.99 (0.98–0.99)	13.2
RVR rate (1/s)	-0.24±0.08	0.98 (0.97–0.99)	15.2	0.38±0.15	0.95 (0.93–0.96)	21.5

CoV- coefficient of variation, CI- confidence interval, ICC- intraclass correlation coefficient, RVCR—right ventricle circumferential strain, RVGLS—right ventricle global longitudinal strain, RVR—right ventricle radial strain, SD- standard deviation.

## Discussion

This is the first study to describe in detail cardiovascular magnetic resonance—derived right ventricular longitudinal, circumferential and radial strain and strain rates in children with hypertrophic cardiomyopathy and to relate these parameters to LVOT obstruction and fibrosis assessed with late gadolinium enhancement. We demonstrated that there was a substantial reduction in RV displacement parameters in obstructive childhood HCM as compared to non-obstructive childhood HCM. Also, few indices of RV mechanics were decreased in children with LGE in comparison to children with HCM and no fibrosis on CMR.

Over the last 50 years, awareness regarding the characterization of HCM has evolved dramatically in terms of phenotypic expression, pathophysiology, and clinical course [19–21]. It is reasonable that the right ventricle may participate in the disease due to the extension of myopathic processes and/or because right and left ventricles share structurally hypertrophied interventricular septum [22]. Previous experimental and clinical studies indicate that the septum is ‘the lion of the right ventricular function’ and the fiber orientation and septal architecture and function are essential for RV ejection and suction for rapid filling [23]. The elevation in LV filling pressures in HCM is transmitted to the left atrium, pulmonary circulation, subsequently to the right atrium and right ventricle, causing pressure overload and decreased performance. Expectedly, in the present study, despite normal RV ejection fraction, abnormal RV deformation was seen in children with LV HCM phenotype proving RV involvement in the myopathic process. Ventricular interaction is an expression of close anatomic association between the two ventricles, which are encircled by common muscle fibers, share a septal wall, and are enclosed within the pericardium. This is emphasized in our study by the association between the deterioration of RV deformation and the aggressiveness of LV phenotype determined by the magnitude of hypertrophy, severity of LVOT obstruction and LV fibrosis.

CMR is a reference standard method for evaluating RV function for diagnostic, prognostic, and therapeutic implications. The excellent signal-to-noise ratio between the myocardium and the blood pool allows for reliable volumetric analysis without geometric assumptions. CMR-FT has been a new less demanding technology for quantifying ventricular mechanics in comparison to the tagging technique [24]. It has been successfully used to demonstrate RV dysfunction in congenital heart disease, pulmonary hypertension and arrhythmogenic RV cardiomyopathy with good interstudy and inter-observer reproducibility [25–27]. In our study, we obtained a satisfactory reproducibility of RV deformation parameters assessed in children with HCM.

The data on RV deformation assessed by CMR-FT in adult HCM population are very limited, and no such work has been performed in childhood HCM. In an echocardiographic study, Cincin et al. reported significant impairment of RV function and 2D-STE-based strain of the RV free wall basal segment in HCM adults in comparison to control subjects [28]. Moreover, Badaran et al. used vector velocity imaging and showed severe displacement incorrectness at all levels of RV in HCM adults [22]. Our study is the first to demonstrate an impairment of RV mechanics in the childhood HCM population by means of CMR-FT. Our results of CMR-FT-derived RVGLS are slightly higher than the values obtained with echocardiography in adults [29]. Interestingly, in the study by Cincin et al., unlike in our study, LVOT obstruction in HCM was not associated with the impairment of RV mechanics [28]. Our study contradicts that finding and represents a completely different view. This can be explained by modality and population age differences. In the present study, the deformation abnormalities were the first sign of the disease as no difference in RVEF was found between children with and those without LVOT obstruction. Further studies are necessary to determine accurate relations of LVOT obstruction and RV mechanics, particularly in the paediatric population.

### Limitations of the study

The limitations of our study are mostly inherited by its design. This is a retrospective study with a relatively small sample size. It must also be noted that the study was conducted over an extended period of time, and there has been advancement in CMR technology. For example, while mapping techniques are now important to the diagnosis of HCM, they were not applied to our cohort. Large, prospective, multicentre studies are needed to validate our results and to provide further evidence that RV dysfunction is associated with a disease state and poor prognosis of HCM in young subjects.

## Conclusions

This work helps to better understand the RV malfunction in HCM children. All indices of RV myocardial mechanics in HCM juveniles were globally compromised when LVOTO was also present. Moreover, the degree of LVOT obstruction, rather than fibrosis, appeared to be a factor associated with the reduction of RV mechanics.

## Supporting information

S1 Data (XLSX)

## Abbreviations

BSA: body surface area
CMR: cardiovascular magnetic resonance
FT: feature tracking
HCM: hypertrophic cardiomyopathy
LAA: left atrial area
LGE: late gadolinium enhancement
LGE%LV: amount of late gadolinium enhancement as a percentage of left ventricular mass
LV: left ventricle
LVEDV: left ventricular end-diastolic volume
LVEDVI: left ventricular end-diastolic volume index
LVEF: left ventricular ejection fraction
LVESV: left ventricular end-systolic volume
LVESVI: left ventricular end-systolic volume index
LVM: left ventricular mass
LVMI: left ventricular mass index
LVOT: left ventricular outflow tract
LVOTO: left ventricular outflow tract obstruction
LVSV: left ventricular stroke volume
LVSVI: left ventricular stroke volume index
MR: mitral regurgitation
RV: right ventricle
RVCR: right ventricular circumferential strain
RVEDV: right ventricular end-diastolic volume
RVEDVI: right ventricular end-diastolic volume index
RVEF: right ventricular ejection fraction
RVESV: right ventricular end-systolic volume
RVESVI: right ventricular end-systolic volume index
RVGLS: right ventricular global longitudinal strain
RVR: right ventricular radial strain
RVSV: right ventricular stroke volume
RVSVI: right ventricular stroke volume index
SSFP: steady-state free precession
STE: speckle-tracking echocardiography
